# Automated measurement of iris surface smoothness using anterior segment optical coherence tomography

**DOI:** 10.1038/s41598-021-87954-w

**Published:** 2021-04-19

**Authors:** Mohammad Zarei, Tahereh Mahmoudi, Hamid Riazi-Esfahani, Behnam Mousavi, Nazanin Ebrahimiadib, Mehdi Yaseri, Elias Khalili Pour, Hossein Arabalibeik

**Affiliations:** 1 Retina Service, Farabi Eye Hospital, Tehran University of Medical Sciences, Qazvin Square, South Kargar Street, Tehran, 1336616351 Iran; 2 Department of Medical Physics and Biomedical Engineering, Tehran University of Medical Sciences and Research Center for Science and Technology in Medicine, Tehran, Iran; 3 Department of Epidemiology and Biostatistics, School of Public Health, Tehran University of Medical Sciences, Tehran, Iran

**Keywords:** Medical imaging, Tomography, Diagnostic markers

## Abstract

Fuchs uveitis (FU) is a chronic and often unilateral ocular inflammation and characteristic iris atrophic changes, other than heterochromia, are common in FU and are key to the correct diagnosis in many cases. With the advent of anterior segment optical coherence tomography (AS-OCT), some investigators attempted to quantitatively study these atrophic changes; mostly by introducing various methods to measure iris thickness in AS-OCT images. We aimed to present an automated method in an observational case series to measure the smoothness index (SI) of the iris surface in AS-OCT images. The ratio of the length of the straight line connecting the most peripheral and central points of the anterior iris border (in nasal and temporal sides) to the actual length of this border on AS-OCT images, was defined as SI. In a uveitis referral center, twenty-two eyes of 11 patients with unilateral Fuchs uveitis (FU) (7 female) and 22 eyes of 11 healthy control subjects underwent AS-OCT imaging. Image J and a newly developed MATLAB algorithm were used for manual and automated SI measurements, respectively. Agreement between manual and automated measurements was evaluated with Bland–Altman analysis and interclass correlation coefficient. The inter-eye difference of SI was compared between the FU group and the control group. Automated mean overall SI was 0.868 ± 0.037 and 0.840 ± 0.039 in FU and healthy fellow eyes, respectively (estimated mean difference =  − 0.028, 95% CI [− 0.038, − 0.018], *p* < 0.001). Bland- Altman plots showed good agreement between two methods in both healthy and FU eyes. The interclass correlation coefficient between the manual and automated measurements in the FU and healthy fellow eyes was 0.958 and 0.964, respectively. The inter-eye difference of overall SI was 0.029 ± 0.015 and 0.012 ± 0.008 in FU group and control group, respectively (*p* = 0.01). We concluded that the automated algorithm can rapidly and conveniently measure SI with results comparable to the manual method.

## Introduction

Fuchs uveitis (FU) is a chronic and often unilateral ocular inflammation that accounts for about 1 to 6% of all patients referred to uveitis clinics^1–3^. Despite the lack of definite criteria, diagnosis is usually based on the presence of some of the following characteristics: fine stellate keratic precipitates (Fuchs KPs), mild anterior chamber reaction, iris atrophic changes (including heterochromia), vitreous cells, and debris, posterior subcapsular cataract, glaucoma, and a characteristic absence of macular edema and posterior synechiae. However, the presenting specific signs and symptoms may be subtle and misdiagnosis of FU remains a common clinical problem^4–7^.

Even though heterochromia is a common characteristic finding in patients of Western European descent, it is much less common in patients from other ethnicities. However, regardless of ethnic differences, characteristic iris atrophic changes, other than heterochromia, are common in FU and are key to the correct diagnosis in many cases^7^. With the advent of anterior segment optical coherence tomography (AS-OCT), some investigators attempted to quantitatively study these atrophic changes,mostly by introducing various methods to measure iris thickness in AS-OCT images. However, the results are mixed^8–10^.

Among atrophic iris changes in FU, is increased smoothness of iris surface (decreased prominence of iris crypts) in the involved eyes. This increased smoothness is best appreciated in comparison to the healthy fellow eye in unilateral cases. Considering the paramount usefulness of this finding in clinical practice, we have recently shown that a qualitative examination of AS-OCT images for this finding has considerable diagnostic potential. A shortcoming of this technique is its qualitative nature. To address this drawback, we have also introduced “smoothness index (SI)”; a quantitative index for iris surface smoothness, and proposed a manual technique to measure this index in AS-OCT images. Smoothness index was defined as the ratio of the length of a straight line connecting the most peripheral and the most central points of the anterior iris border to the actual length of this border^11^. In unilateral FU, shallower iris crypts in the affected eye compared to the healthy fellow eye is expected to lead to a larger SI.

Applying this manual technique on AS-OCT images from 20 patients with unilateral FU and 20 healthy subjects as the control group, showed that it is useful for quantitative documentation of the increased smoothness of the iris surface in the involved eye compared to the healthy fellow eye^12^. However, the manual segmentation of AS-OCT images with ImageJ software which is time-consuming and requires a skillful operator potentially limits the practical usefulness of the SI. To address the limitations we developed an automated algorithm for the calculation of the SI in AS-OCT images. The purpose of the current study is to introduce this automated method and compare its results with the results of the manual method.

## Methods

This study was conducted at Farabi Eye Hospital, Tehran, Iran. The study followed the tenets of the Declaration of Helsinki and was approved by the Tehran University of Medical Sciences Institutional Review Board (IRB) (IR.TUMS.FARABIH.REC.1395.2878, http://ethics.research.ac.ir) and written informed consents were obtained from the patients.

The diagnosis of unilateral FU was made by either of two authors (MZ or NE) and was based on unilateral chronic anterior uveitis, typical Fuchs KPs, absence of posterior synechiae, and absence of macular edema in macular spectral-domain OCT and/or FA. Unilateral presence of open-angle glaucoma or ocular hypertension, posterior subcapsular cataract, vitreous cells or degeneration, a smoother surface of the iris (compared to the healthy fellow eye), and heterochromia were considered supportive, but not necessary for the diagnosis of FU. Exclusion criteria were any abnormal focal or multifocal findings in iris structure (other than nodules), intumescent cataract, consumption of any medication affecting pupillary diameter, and history of ocular trauma, laser treatment, or surgeries on either eye^12^.

To compare the inter-eye difference of the SI in Fuchs patients with healthy subjects, an age-sex matched group of healthy subjects served as the control group. Complete ocular exam and the same imaging were performed for the control group. The inter-eye difference in automated SI was compared between the FU group and the control group.

Eligible subjects underwent AS-OCT scanning (swept-source (SS)-OCT CASIA 2, Tomey, Japan) in the regular day room illumination without prior administration of any mydriatic or miotic drops. Both eyes of each subject were imaged in a single session with the same room illumination. Horizontal B scans centered at the pupillary center, including the iris from 3 o’clock to 9 o’clock were selected for evaluation. During the imaging, subjects were instructed to open their eyes as wide as possible and not to blink. If an artifact was noted, the imaging was repeated until an acceptable image was achieved.

In the FU group, the smoothness index for each eye was calculated by manual and automated methods. In the control group, the smoothness index for each eye was calculated by the automated method.

### Manual calculation of smoothness index

All images were exported to and analyzed with ImageJ (ImageJ version 1.52, NIH, USA) software to manually segment and measure SI. To assess the intra- and inter-reliability value of the manual method, we measured SI in all images twice in separate sessions by two independent raters (E.K and H.R). To measure the lengths, the free-hand tool of ImageJ with 300 percent magnification was used.

**Figure 1:**
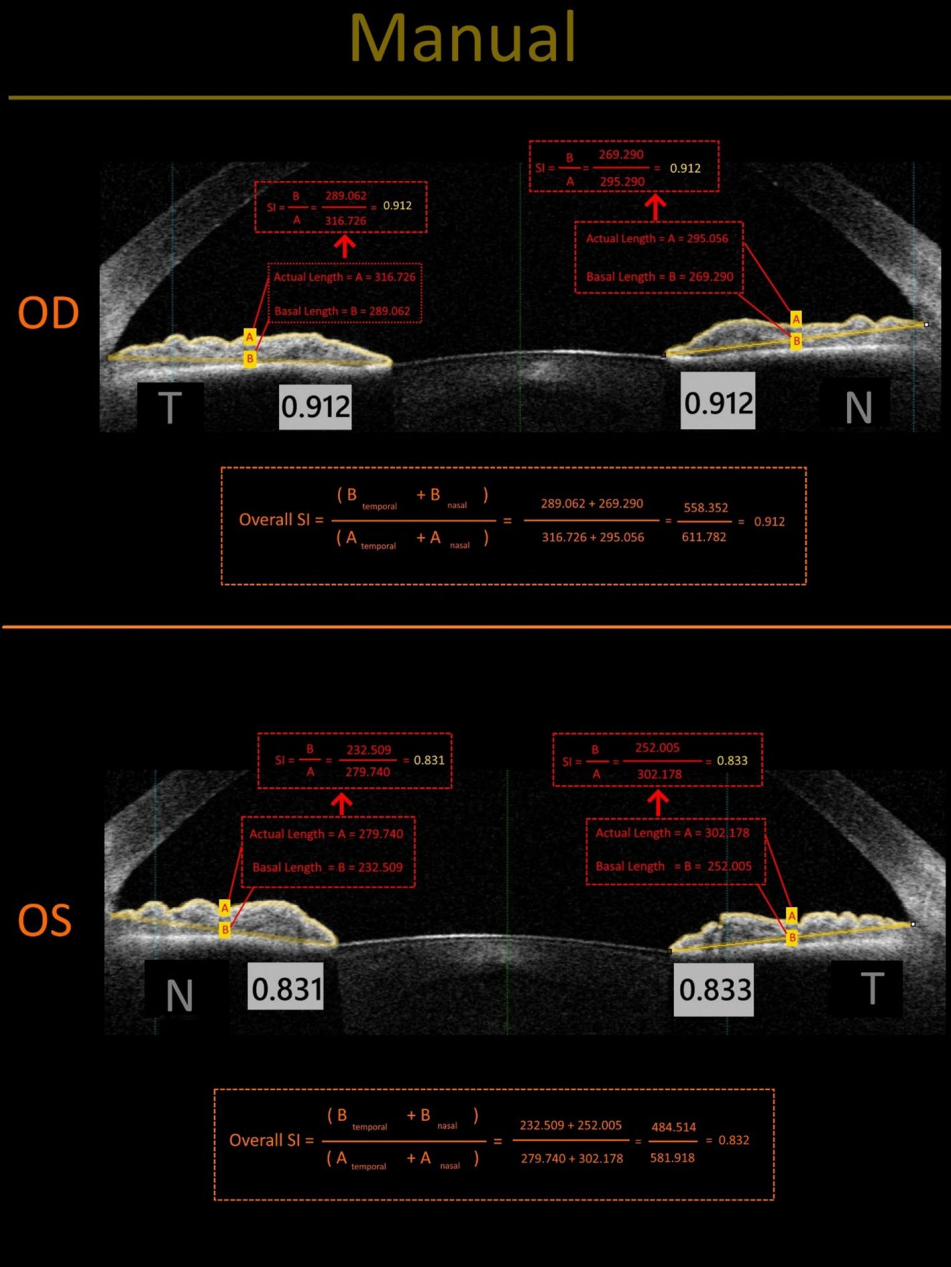
Manual measurement and calculation of the smoothness index. Due to the blunting of the iris crypts, the eye affected by Fuchs uveitis (OD) has a larger SI than the healthy fellow eye (OS).

As we previously proposed, Overall SI was defined as the ratio of the length of a straight line connecting the most peripheral and the most central points of the anterior iris border (in nasal and temporal sides) divided to the actual length of this border (in nasal and temporal sides) Fig. 1.

$$Overall SI= \frac{\mathrm{length of the straight line connecting the most peripheral and the most central point of the iris in nasal side}+\mathrm{length of the same line in the temporal side}}{Actual length of iris border \mathrm{connecting the most peripheral and the most central point in nasal side}+\mathrm{length of the same line in temporal Side}}$$

Hereafter, as a matter of convenience, we will use the term *basal length of anterior iris border* instead of the “length of the straight line connecting the most peripheral and the most central point of the anterior iris border”^12^.

### Automated calculation of smoothness index (SI)

To use the automated method, the operator just needed to select four points on the AS-OCT image: the most peripheral and the most central points of the anterior iris border (in nasal and temporal sides).

**Figure 2:**
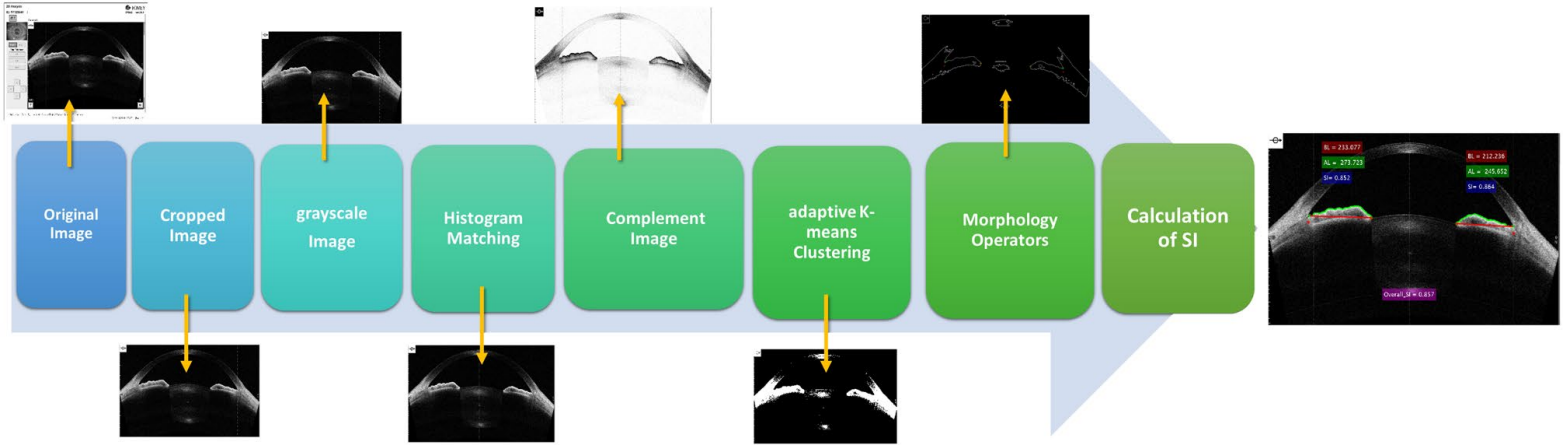
Block diagram of the proposed automated method.

In this study, all of the image processing algorithms for automated calculation of the SI were implemented using Matlab software R2019a (Mathworks, Inc., Natick, MA). The development of the proposed automated method consists of two steps: first, the image enhancement, and second, the calculation of the SI. The block diagram of the method is shown in Fig. 2.

**Figure 3:**
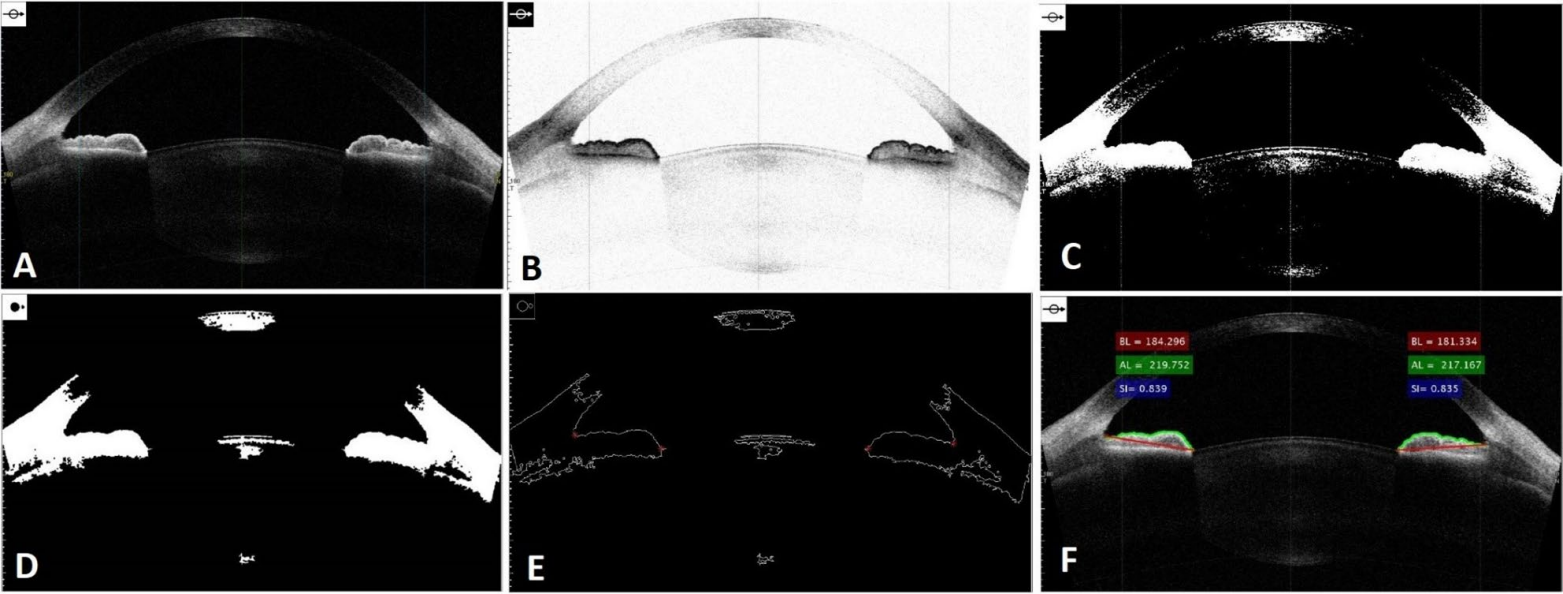
Image processing to calculate the SI index: (**A**) original image (**B**) complemented image (**C**) binarized image (**D**) binary image after applying morphology operator (**E**) delineating the margins (**F**) results of the calculation of SI (BL: basal length, AL: actual length, SI: smoothness index).

The original resolution of the images was $1000 \times 1414$ pixels. These images were cropped to $629\times 1102$ pixels and converted to grayscale. In this algorithm, the original image and a reference image is used to produce the output image. The reference image was the image with better quality and contrast than the other images. For quality equalization and enhancement of the images, the histogram of the reference image was approximately matched with the histogram of the reference image and then the output image was used for the rest of the processing. This histogram matching was applied to each image separately Fig. 3A.

For better visualization, we used the complement (negative-mode) of the images Fig. 3B. In the complement of a grayscale or color image, each pixel value is subtracted from the maximum pixel value supported by the class (or 1.0 for double-precision images). The difference is used as the pixel value in the output image. In the output image, dark areas become lighter and light areas become darker.

To determine the anterior border of the iris, we used the “adaptive K-means clustering” segmentation algorithm^13^. Adaptive K-means clustering, partitions image into K classes of gray levels. Since we wanted to segregate the anterior margin of the iris in the image from the background, we needed two classes. For all the images, we considered pixels of the anterior iris border as the first-class and the rest of the image pixels as the background, so the output image after adaptive K-means clustering was a binarized image Fig. 3C.

Then, morphology operator algorithms were used to eliminate small areas of defects in the iris border in the binarized image to produce a continuous iris border Fig. 3D^14^. Thereafter, the peripheral borders of the objects in the image were extracted to delineate the margins of the iris Fig. 3E.

To calculate the SI, the automated algorithm must perform two measurements on the final output image: 1- measuring the basal length of the anterior iris border 2-measuring the actual length of the anterior iris borderFig. 3F.

**Figure 4:**
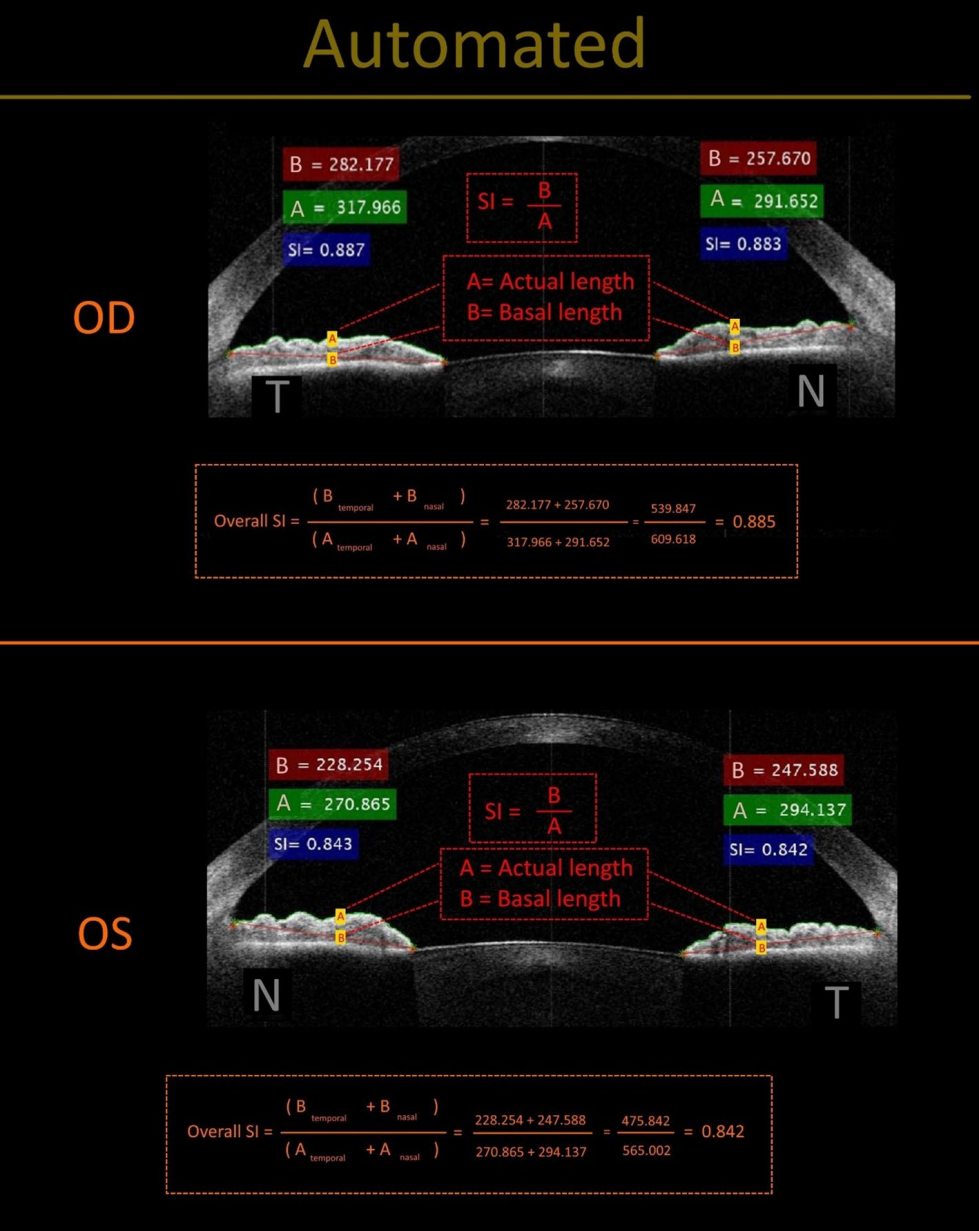
Results of automated calculation of the smoothness index for the same patients as in Fig. 1.

To calculate the basal length of the anterior iris border, we used the shortest path function proposed by Steve Eddins^15^. Using this algorithm, we calculated the distance between one point selected by the user and all of the non-zero pixels located in the other areas and constructed a distance matrix for this point, then this stage was repeated for the second point selected by the user. By adding two obtained distance matrices, the local minimum which is the shortest path was returned. To calculate the actual length of the anterior iris border, we obtained Euclidean distance between the two points mentioned above. Figure 4 illustrates calculating the SI using the automated method for the same patient as in Fig. 1.

To assess the intra- and inter-rater repeatability and reproducibility value of the automated method, we automatically measured SI twice, by two independent raters.

The code for the automated measurement is presented in supplement 1.

### Statistical analysis

We used mean, standard deviation, median, and range, frequency, and percentage to describe variables. In the FU group, to compare mean SI between Fuchs eyes and healthy fellow eyes, a generalized estimating equation (GEE) was used.

In the FU group, Bland–Altman limits of agreement between manual and automated measurements were calculated for healthy fellow eyes and Fuchs eyes separately. Bland–Altman plots were used to graphically present the agreement between manual and automated methods in calculating the overall SI. Interclass correlation coefficients between the two methods were also calculated.

The mean age between the FU group and the control group was compared with the t-test. Mann–Whitney test was used to compare the absolute inter-eye difference between the FU group and the control group.

All statistical analysis was performed by SPSS (IBM Corp. Released 2013. IBM SPSS Statistics for Windows, Version 22.0. Armonk, NY: IBM Corp.). *P* value less than 0.05 was considered statistically significant.

## Results

Twenty-two eyes from 11 phakic patients with unilateral FU were enrolled in this study (7 female and 4 male). The mean age of patients was 39.55 ± 12.43 years (range: 29–69). Demographic and clinical features are summarized in Table 1.

**Table 1:** Demographic and clinical features of Fuchs patients.

Patients	Age (years)	Gender	Eye	Involvement by FU	Snellen BCVA	Fuchs KPs	Heterochromia	Posterior subcapsular cataract	Vitritis	Vitreous degeneration
Patient 1	69	Female	OD	No	20/32	No	No	No	No	No
			OS	Yes	20/200	Yes	No	Yes	Yes	No
Patient 2	29	Female	OD	No	20/20	No	No	No	No	No
			OS	Yes	20/32	Yes	No	Yes	Yes	Yes
Patient 3	32	Female	OD	No	20/20	No	No	No	No	No
			OS	Yes	CF	Yes	No	Yes	Yes	Yes
Patient 4	33	Male	OD	Yes	20/32	Yes	No	No	Yes	No
			OS	No	20/20	No	No	No	No	No
Patient 5	35	Male	OD	No	20/20	No	No	No	No	No
			OS	Yes	CF	Yes	No	Yes	No	No
Patient 6	30	Female	OD	No	20/20	No	No	No	No	No
			OS	Yes	20/32	Yes	No	Yes	Yes	Yes
Patient 7	33	Female	OD	No	20/20	No	No	No	No	No
			OS	Yes	20/50	Yes	No	Yes	Yes	No
Patient 8	59	Female	OD	No	20/20	No	No	No	No	No
			OS	Yes	20/100	Yes	No	Yes	Yes	Yes
Patient 9	39	Male	OD	Yes	20/20	Yes	No	Yes	No	No
			OS	No	20/20	No	No	No	No	No
Patient 10	40	Male	OD	No	20/20	No	No	No	No	No
			OS	Yes	20/40	Yes	No	Yes	Yes	No
Patient 11	36	Female	OD	No	20/20	No	No	No	No	No
			OS	Yes	20/100	Yes	No	No	Yes	Yes

*CF* counting fingers, *BCVA* best-corrected visual acuity, *KP* keratic precipitate.

Twenty-two phakic eyes from 11 healthy subjects were chosen as the control group. Female: male ratio in the control group was the same as the case group (7:4). The mean age in the control group was 40.27 ± 13.36 (range: 29–73) years which was not statistically different from FU group (*p* = 0.897).

Automated and manual SI for temporal, nasal, and overall iris were measured for all eyes (in both control and FU groups) and presented in supplement file 2. Supplement file 3 contains the AS-OCT images of all eyes (both control and FU groups) with automated nasal, temporal, and overall SI measurements.

Manual mean overall SI was 0.861 ± 0.041 (range: 0.778–0.918) in controls. Manual mean overall SI was 0.841 ± 0.043 (range: 0.768–0.892) and 0.873 ± 0.039 (range: 0.798–0.913) in healthy fellow eyes and Fuchs eyes, respectively (estimated mean difference = − 0.033, 95% CI [− 0.048, − 0.017], *p* < 0.001).

**Figure 5:**
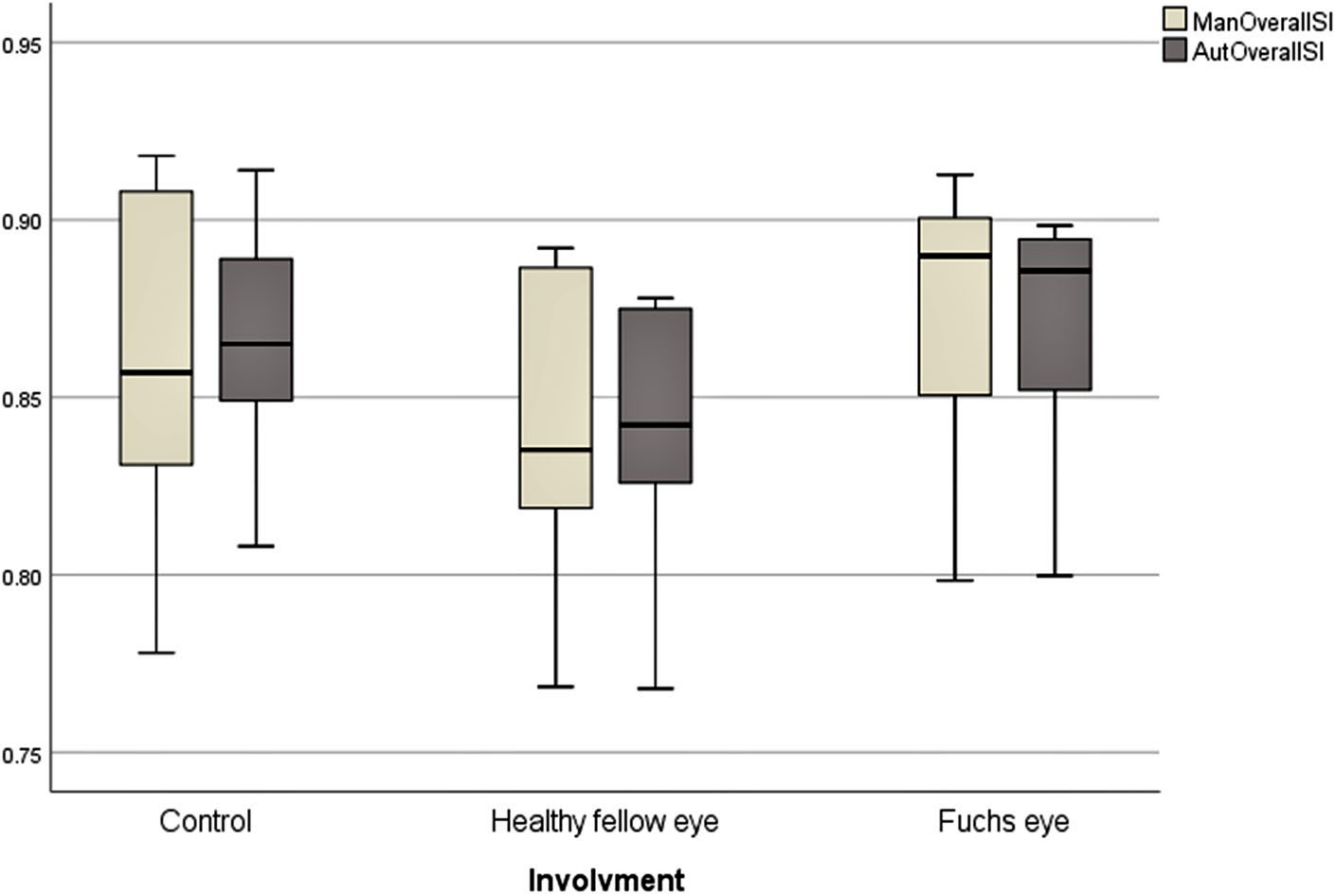
Manual and automated overall SIs in controls, healthy fellow eyes (healthy), and Fuchs uveitis (FU) eyes in the FU group.

Automated mean overall SI was 0.863 ± 0.032 (range: 0.808 to 0.914) in controls. Automated mean overall SI was 0.840 ± 0.039 (range: 0.768–0.878) and 0.868 ± 0.037 (range: 0.800 to 0.898) in healthy fellow eyes and Fuchs eyes, respectively (estimated mean difference = − 0.028, 95% CI [-0.038, -0.018], *p* < 0.001). Table 2 presents a detailed comparison of mean SIs (manual and automated) between healthy fellow eyes and Fuchs eyes in the FU group. Figure 5 presents a graphical comparison of manual and automated overall SIs in FU and healthy fellow eyes in the FU group.

**Figure 6:**
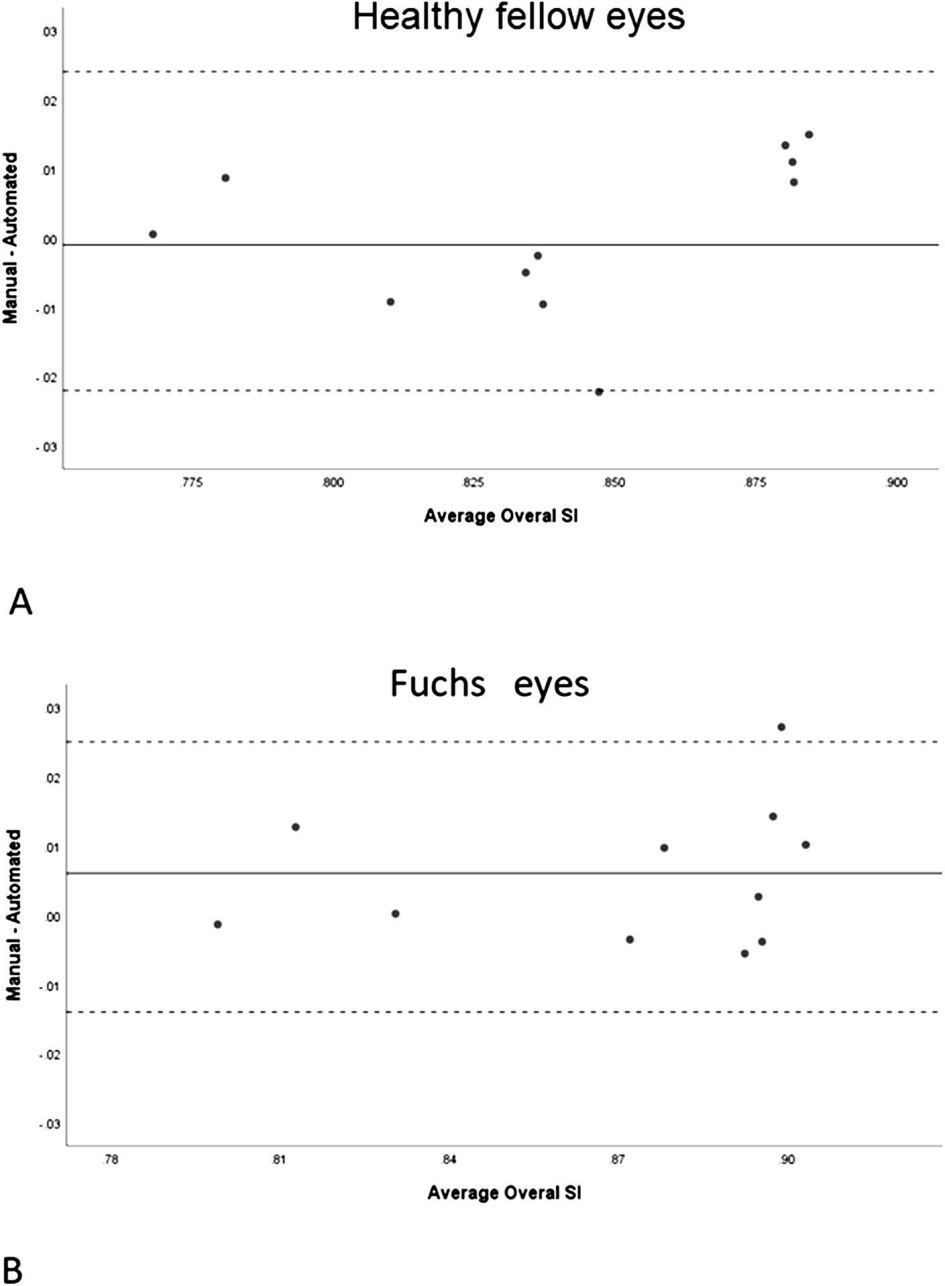
Bland Altman plots for manual and automated measurement of average overall SI in healthy fellow eyes (**A**) and Fuchs eyes (**B**) in the Fuchs group.

In the control group, the limits of agreement between manual and automated methods for measuring overall SI were − 0.04 to 0.04. In the FU group, limits of agreement between manual and automated methods for measuring overall SI were − 0.02 to 0.02 and − 0.01 to 0.03 in healthy fellow eyes and Fuchs's eyes, respectively. Table 3 shows the limits of agreement between manual and automated measurements of the SI in the FU group (in the temporal iris, nasal iris, and overall iris). Bland–Altman plots to present the agreement between manual and automated measurements of the overall SI in healthy fellow eyes and FU eyes in the FU group are shown in Fig. 6. The interclass correlation coefficient between manual and automated measurements in healthy fellow eyes and FU eyes was 0.964 and 0.958, respectively.

Table 4 shows the mean inter-eye difference of automated temporal, nasal, and overall SI in the FU group and the control group. The mean inter-eye difference of overall (0.029 ± 0.015 vs. 0.012 ± 0.008) and nasal (0.032 ± 0.023 vs. 0.013 ± 0.01) automated SI were significantly higher in FU group in comparison with control group (*p* = 0.01 and *p* = 0.03, respectively). The mean inter-eye difference of temporal automated SI was also higher in the FU group (0.027 ± 0.02) in comparison with the control group (0.019 ± 0.012), although it did not reach statistical significance (*p* = 0.41).

**Figure 7:**
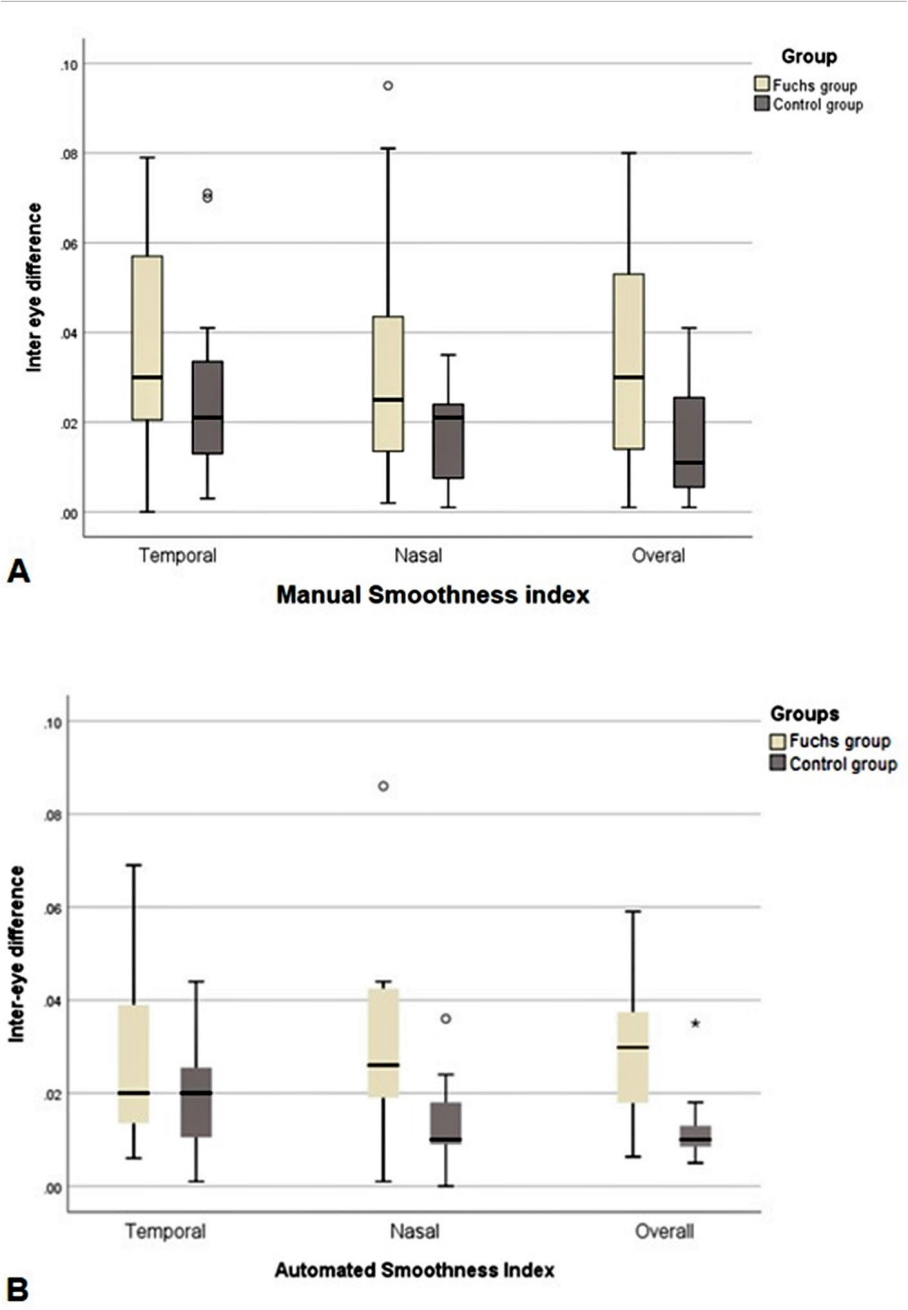
Box-plots comparing the temporal, nasal, and overall inter-eye difference between the FU and the control groups by both manual (**A**) and automated (**B**) methods.

Figure 7 compares the temporal, nasal, and overall inter-eye difference of SI between the FU group and the control group.

The intra-rater reliability of the manual method based on two-way random ICC (intracluster coefficient) was found to be 0.872 (95% CI 0.722–0.945). The ICC for inter-rater reliability of the manual method was 0.799 (95% CI 0.263–0.932).

Meanwhile, in the automated method, the intra-rater reliability based on two-way random ICC (intracluster coefficient) was found to be 0.988 (95% CI 0.978–0.993). The ICC for inter-rater reliability of the automated method was 0.959 (95% CI 0.823–0.984). Based on these results it seems that the automated method has high inter and intra-rater reliability value.

## Discussion

Diffuse iris atrophy is among the most characteristic findings in the eyes affected by FU^3^. However, in some cases, these changes are subtle and difficult to appreciate on clinical examination. On the other hand, previous studies on the iris thickness changes in AS-OCT images have reported mixed results^8–10^.

In most cases of FU, iris atrophy leads to blunting of the iris crypts and the affected iris appears to be smoother than the iris of the healthy fellow eye. However, the clinical usefulness of the iris surface smoothness as a key to diagnosing FU is highly dependent on the examiner’s expertise and vigilance to search for this finding. This led us to define an index (SI) to quantify this feature in AS-OCT images. We found that manual measurement of the SI may serve as a useful technique in the diagnosis of unilateral FU^12^. However, manual measurement is time-consuming and needs a skillful operator. We found that on average, an experienced operator needs about three minutes to manually measure and calculate the SI. Moreover, in the manual method, the operator has to accurately identify the anterior margin of the iris in the magnified AS-OCT image, and this may lead to error, especially in cases with deep iris crypts or crypts of complex architecture. Some operators may also find the mental focus needed for the whole process a challenge. Considering these limitations, we developed an automated method to make the SI measurement more user-friendly and therefore more applicable in clinical practice and research. With this automated method, from the moment the operator starts to select four points on the iris AS-OCT image -the most peripheral and central points of the anterior iris border- it takes about 5–10 s to reach the SI,the advantage in time and convenience is obvious. Our previous experience on manual measurements of SI by ImageJ software showed that the manual method has potential drawbacks like being a time-consuming process and potentially lower reliability and repeatability, most probably due to inability to be well-focused inline with iris border by mouse or a digital pen^12^. Interestingly, based on two-way random ICC (intracluster coefficient) analysis in the automated method in the current study, it seems that the automated method has high inter and intra-rater reliability values.

Whatever the AS-OCT device, this automated technique can be applied to any image of adequate quality. Our results showed that temporal, nasal, and overall iris of the FU eyes have a significantly larger SI compared to the healthy fellow eyes. This difference was seen in the automated as well as the manual measurements Table 2 and Fig. 5. In both subgroups of the eyes, the results from the automated method showed good agreement with the results from the manual method Table 3 and Fig. 6.

**Table 2:** Comparison of mean manual and automated SIs between Fuchs eyes and healthy fellow eyes in Fuchs patients.

		Involvement		Difference	95% CI		*P*†
		Healthy	FU		Lower	Upper	
Manual temporal SI	Mean ± SD	0.84 ± 0.04	0.875 ± 0.045	− 0.036	0.053	− 0.019	< 0.001
	Median (range)	0.839 (0.77–0.883)	0.896 (0.793–0.917)				
Automated temporal SI	Mean ± SD	0.841 ± 0.041	0.869 ± 0.044	− 0.027	− 0.039	− 0.016	< 0.001
	Median (range)	0.842 (0.761–0.883)	0.887 (0.779–0.906)				
Manual Nasal SI	Mean ± SD	0.841 ± 0.051	0.870 ± 0.04	− 0.029	− 0.049	− 0.009	0.005
	Median (range)	0.831 (0.764–0.903)	0.881 (0.803–0.92)				
Automated Nasal SI	Mean ± SD	0.838 ± 0.041	0.867 ± 0.036	− 0.028	− 0.044	− 0.013	< 0.001
	Median (range)	0.843 (0.76–0.88)	0.883 (0.804–0.917)				
Manual overall SI	Mean ± SD	0.841 ± 0.043	0.873 ± 0.039	− 0.033	− 0.048	− 0.017	< 0.001
	Median (range)	0.835 (0.768–0.892)	0.890 (0.798–0.913)				
Automated overall SI	Mean ± SD	0.84 ± 0.039	0.868 ± 0.037	− 0.028	− 0.038	− 0.018	< 0.001
	Median (range)	0.842 (0.768–0.878)	0.886 (0.8–0.898)				

^†^Based on GEE analysis.

*SI* smoothness index, *Healthy* healthy fellow eyes, *FU* Fuchs uveitis eyes.

**Table 3:** Limits of agreement between manual and automated measurements in Fuchs eyes and healthy fellow eyes in the Fuchs group.

	Control eyes			Healthy fellow eyes			Fuchs eyes		
	Mean diff	LoA		Mean diff	LoA		Mean diff	LoA	
		Lower	Upper		Lower	Upper		Lower	Upper
Temporal SI	0.00	− 0.06	0.06	0.00	− 0.02	0.02	0.00	− 0.02	0.03
Nasal SI	0.00	− 0.04	0.04	0.00	− 0.03	0.04	0.00	− 0.02	0.02
Overall SI	0.00	− 0.04	0.04	0.00	− 0.02	0.02	0.01	− 0.01	0.03

*SI* smoothness index, *LoA* limits of agreement.

Compared to the control group, the inter-eye difference of automated nasal and overall SI was significantly higher in patients with unilateral FU. Although the inter-eye difference in automated temporal SI was also higher in the FU group than the control group, it did not reach the limit of the statistical significance; probably due to the small sample size Table 4 and Fig. 7.

**Table 4:** Absolute inter-eye difference of automated temporal, nasal and overall SI in the Fuchs group and the control group.

Inter-eye difference of SI		Group		Ratio	*P*†
		Fuchs	Control		
Temporal	Mean ± SD	0.027 ± 0.02	0.019 ± 0.012	1.42	0.41
	Median (range)	0.02 (0.006–0.069)	0.02 (0.001–0.044)		
Nasal	Mean ± SD	0.032 ± 0.023	0.013 ± 0.01	2.46	0.03
	Median (range)	0.026 (0.001–0.086)	0.01 (0–0.036)		
Overall	Mean ± SD	0.029 ± 0.015	0.012 ± 0.008	2.42	0.01
	Median (range)	0.03 (0.006–0.059)	0.01 (0.005–0.035)		

^†^Based on Mann–Whitney test.

*SI* smoothness index.

Most iris changes in FU are characteristically diffuse. Therefore, an increase in SI in other meridians of the iris is expected to be observed as well. This remains to be shown in future studies.

Considering that FU is chronic uveitis, it is impossible to pinpoint the onset of inflammation in most cases. However, longitudinal studies may help to find whether or not the smoothness of the iris surface changes over the course of FU.

Some investigators, probably in an effort to enhance the reproducibility of measurements, have used miotic drops prior to the acquisition of AS-OCT images^9,10^. However, when we first came up with the idea of smoothness index, our main goal was to find a quantitative equivalent for the blunted iris surface appearance which is observed in routine eye exam of FU patients. Therefore, we decided not to use pharmacologic miosis to obtain images in a condition as similar as possible to the routine iris examination.

The development of an increasing number of ophthalmic imaging modalities has led to an interest in image quantitative analysis. Therefore, the demand for automated analyses of the images is inevitably growing. In addition to FU, iris changes have been investigated qualitatively and quantitatively in AS-OCT images in other diseases (e.g. angle-closure glaucoma) and biomarkers such as the iris curvature and thickness have been extensively studied^16–18^. We introduced a novel biomarker- smoothness index- and developed a method for its automated measurement. This biomarker shows considerable potential clinical relevance in patients with FU, which may be especially useful in cases without heterochromia, as in the current study that none of the enrolled patients had heterochromia. The possible effects of ethnicity, gender, age, and other ocular diseases on the iris surface smoothness are other subjects of interest that can be addressed in future studies with the method we presented here.

On the other hand, layered architecture is present in many ocular structures (e.g. retina and retinal pigment epithelium). This may provide opportunities for variants of the SI to be used for quantitative evaluation of the irregularities which are frequently seen in these layers in pathologic conditions (e.g. diabetes, age-related macular degeneration, vitreoretinal interface disorders …). The automated method that is presented in this study can be modified and adapted for these new potential applications.

One limitation of the current study is that all enrolled patients did not have heterochromia as a helpful sign in the diagnosis of FU. As mentioned, previously, heterochromia is a common characteristic finding in patients of Western European descent, but it is much less common in patients from other ethnicities so we suggest conducting a study on patients with heterochromia.

In conclusion, we presented an automated algorithm for rapid and convenient measurement of the iris surface smoothness in the AS-OCT images. The measurements from this automated method were comparable with the measurements of the manual method in both healthy eyes and Fuchs eyes.

All data generated or analyzed during this study are included in this published article (and its Supplementary Information files).

## Supplementary Information

Supplementary Information
