# Supplementary material for: Automated measurement of iris surface smoothness using anterior segment optical coherence tomography
Source: Sci Rep. 2021 Apr 19;11:8505. doi: 10.1038/s41598-021-87954-w (PMC8055699; doi:10.1038/s41598-021-87954-w)
Supplement: Supplementary file 1 — Supplementary Information [file 41598_2021_87954_MOESM1_ESM.pdf]

Automated Measurement of Iris Surface Smoothness Using Anterior Segment Optical Coherence Tomography

Mohammad Zarei[1], Tahereh Mahmoudi[2], Hamid Riazi-Esfahani[1], Behnam Mousavi[2], Nazanin Ebrahimiadib[1], Mehdi Yaseri[3], Elias Khalili Pour[1, *], Hossein Arabalibeik[2]

1. Retina Service, Farabi Eye Hospital, Tehran University of Medical Sciences, Tehran, Iran

2. Department of Medical Physics and Biomedical Engineering, Tehran University of Medical Sciences and Reasearch Center for Science and Technology in Medicine, Tehran, Iran

3. Department of Epidemiology and Biostatistics, School of Public Health, Tehran University of Medical Sciences, Tehran, Iran

* Correspondence: Elias Khalili Pour

Retina Service, Farabi Eye Hospital, Tehran University of Medical Sciences, Qazvin Square, South Kargar Street, Tehran 1336616351, Iran

Tel +98 9113727471

Email: ekhalilipour@gmail.com

Supplement file 1:

The code for the automated measurement can be downloaded from below link:

https://drive.google.com/file/d/1aApDPYryte2ldViFGPUuoUkt4WplXyKr/view?usp=sharing

Supplement file 2.

Automatically and manually calculated smoothness index for both eyes of each patient in FU group. SI: Smoothness index, FU: Fuchs uveitis

FU patient	Method	OD			OS			FU Eye
		SI			SI			
		**Temporal**	**Nasal**	**Overall**	**Temporal**	**Nasal**	**Overall**	
**#1**	Automated	.872	.875	0.874	.883	.917	0.898	OS
	Manual	.878	.895	0.887	.897	.920	0.908	
**# 2**	Automated	.854	.863	0.858	.906	.888	0.898	OS
	Manual	.826	.847	0.836	.896	.891	0.894	
**# 3**	Automated	.840	.789	0.815	.873	.875	0.874	OS
	Manual	.838	.773	0.806	.871	.868	0.870	

**#4**	Automated	.887	.883	0.886	.842	.843	0.842	OD
	Manual	.912	.912	0.913	.833	.831	0.833	
**#5**	Automated	.894	.897	0.895	.883	.871	0.877	OS
	Manual	.873	.907	0.890	.883	.901	0.892	
**#6**	Automated	.880	.872	0.876	.896	.891	0.894	OS
	Manual	.879	.895	0.887	.909	.881	0.896	
**#7**	Automated	.839	.835	0.837	.845	.816	0.831	OS

	Manual	.849	.814	0.832	.849	.812	0.831	
**#8**	Automated	.833	.842	0.838	.902	.843	0.873	OS
	Manual	.839	.831	0.835	.917	.846	0.883	
**#9**	Automated	.761	.793	0.777	.779	.836	0.807	OD
	Manual	.770	.801	0.785	.796	.844	0.819	
**#10**	Automated	.775	.760	0.768	.796	.804	0.800	OS
	Manual	.771	.764	0.768	.793	.803	0.798	

**#11**	Automated	.876	.880	0.878	.896	.884	0.891	OS
	Manual	.870	.903	0.886	.916	.890	0.905	

Automatically and manually calculated smoothness index for both eyes in control group. SI: Smoothness index.

Control	Method	OD			OS		
		SI			SI		
		Temporal	Nasal	Overall	Temporal	Nasal	Overall
**#1**	Automated	.866	.889	.877	0.896	.881	.889
	Manual	.895	0.895	0.896	0.874	0.894	0.884
**# 2**	Automated	.913	.909	.911	.898	.889	.893
	Manual	0.914	0.923	0.918	0.927	0.897	0.912
**# 3**	Automated	.911	.904	.908	.915	.914	.914
	Manual	0.913	0.907	0.910	0.933	0.886	0.911
**#4**	Automated	.800	.838	.818	.787	.828	.808
	Manual	0.790	0.825	0.807	0.720	0.846	0.778
**#5**	Automated	.867	.885	.875	.891	.888	.889

	Manual	0.922	0.900	0.912	0.896	0.922	0.908
**#6**	Automated	.855	.865	.859	.811	.807	.809
	Manual	0.871	0.873	0.872	0.800	0.869	0.831
**#7**	Automated	.822	.815	.818	.879	.869	.874
	Manual	0.858	0.859	0.859	0.845	0.850	0.848
**#8**	Automated	.852	.879	.865	.875	.857	.865
	Manual	0.814	0.849	0.831	0.837	0.876	0.856
**#9**	Automated	.911	.837	.870	.811	.841	.824
	Manual	0.824	0.800	0.812	0.827	0.784	0.805
**#10**	Automated	.850	.848	.849	.849	.864	.857
	Manual	0.852	0.851	0.852	0.865	0.886	0.847
**#11**	Automated	.857	.869	.863	.836	.869	.852

	Manual	0.793	0.880	0.832	0.834	0.886	0.858

Supplement file 3. Anterior segment OCT images of all eyes with corresponding automatically calculated nasal, temporal, and overall SIs in both FU group and control group. FU: Fuchs Uveitis, SI: Smoothness index

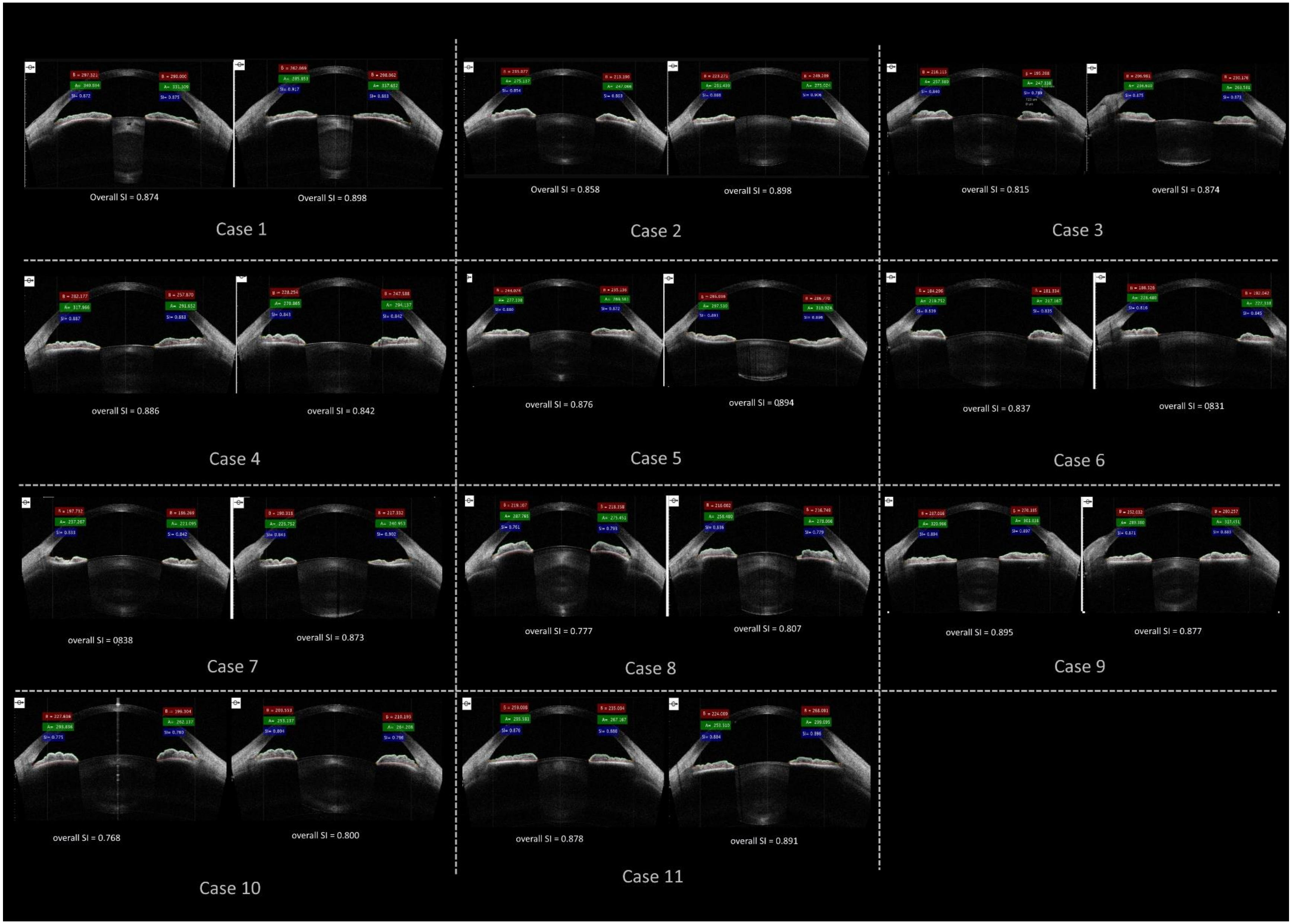
Overall SI = 0.874
Overall SI = 0.898
Case 1
Overall SI = 0.858
overall SI = 0.898
Case 2
overall SI = 0.815
overall SI = 0.874
Case 3
overall SI = 0.886
overall SI = 0.842
Case 4
overall SI = 0.876
overall SI = 0894
Case 5
overall SI = 0.837
overall SI = 0831
Case 6
overall SI = 0838
overall SI = 0.873
Case 7
overall SI = 0.777
overall SI = 0.807
Case 8
overall SI = 0.895
overall SI = 0.877
Case 9
overall SI = 0.768
overall SI = 0.800
Case 10
overall SI = 0.878
overall SI = 0.891
Case 11

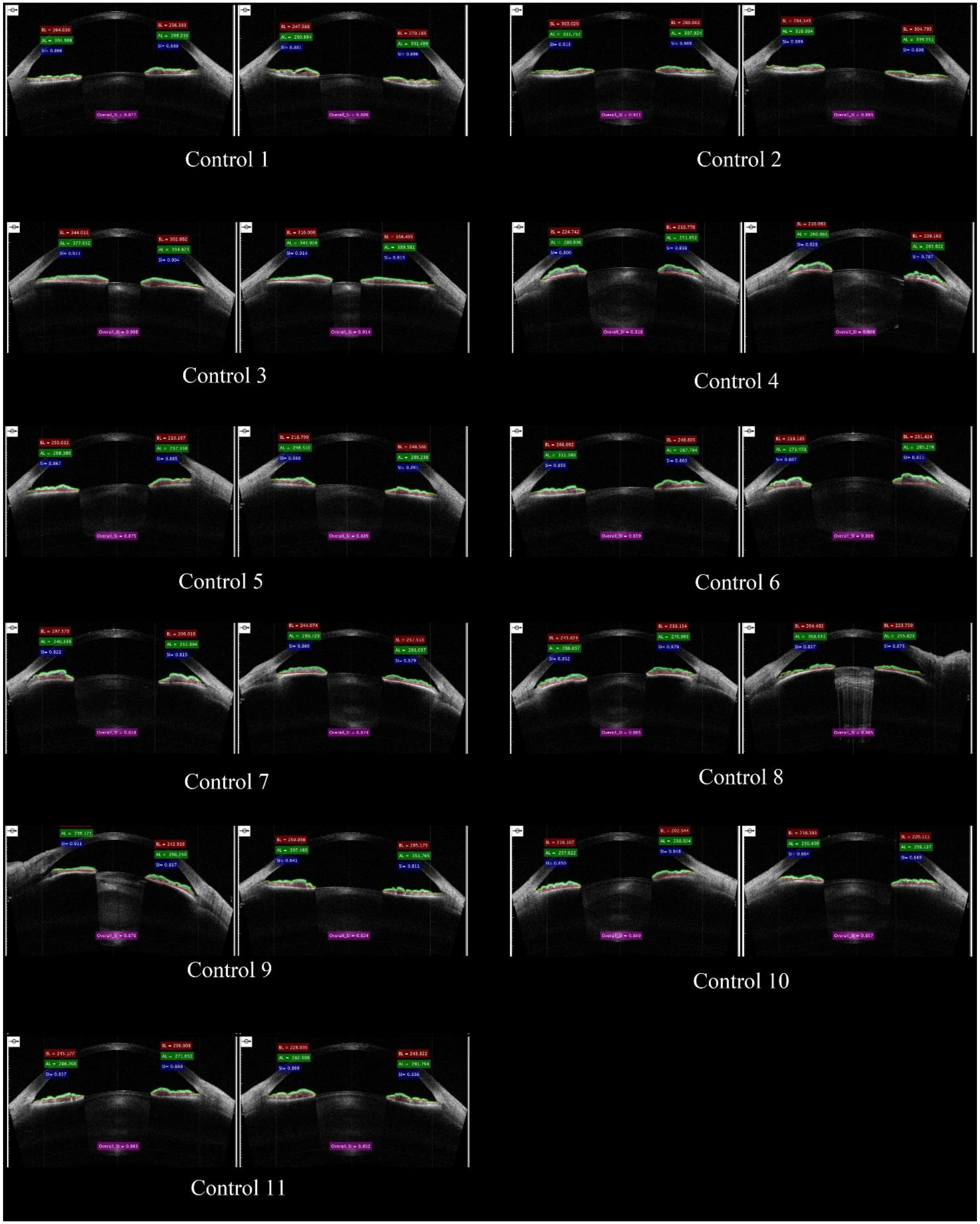
Control 1
Control 2
Control 3
Control 4
Control 5
Control 6
Control 7
Control 8
Control 9
Control 10
Control 11
